# Successful implementation of best medical treatment for patients with asymptomatic carotid artery stenosis within a randomized controlled trial (SPACE-2)

**DOI:** 10.1186/s42466-021-00153-w

**Published:** 2021-10-19

**Authors:** Tilman Reiff, Hans-Henning Eckstein, Ulrich Mansmann, Olav Jansen, Gustav Fraedrich, Harald Mudra, Werner Hacke, Peter Arthur Ringleb, for the SPACE-2 study group, D. Böckler, M. Böhm, H. Brückmann, E. S. Debus, J. Fiehler, W. Lang, K. Mathias, E. B. Ringelstein, J. Schmidli, R. Stingele, R. Zahn

**Affiliations:** 1 Department of Neurology, University Hospital of Heidelberg, Im Neuenheimer Feld 400, 69120 Heidelberg, Germany; 2 Department for Vascular and Endovascular Surgery, Klinikum Rechts Der Isar, Technical University of Munich, Munich, Germany; 3 Institute of Medical Informatics, Biometry and Epidemiology, Ludwig-Maximilians-Universität München, Munich, Germany; 4 Department of Radiology and Neuroradiology, UKSH Campus Kiel, Kiel, Germany; 5 Department of Vascular Surgery, University Hospital of Innsbruck, Innsbruck, Austria; 6 Department of Cardiology, Klinikum Neuperlach, München KlinikMunich, Germany

**Keywords:** Asymptomatic carotid artery stenosis, Primary prevention, Carotid endarterectomy, Carotid artery stenting, Best medical treatment, Disease-free survival, Epidemiology, Prospective study

## Abstract

**Background:**

Asymptomatic carotid artery stenosis (ACS) can be treated with carotid endarterectomy (CEA), carotid artery stenting (CAS), or best medical treatment (BMT) only. For all treatment options, optimization of vascular risk factors such as arterial hypertension, hyperlipidemia, smoking, obesity, and insufficient physical activity is essential. Data on adherence to BMT and lifestyle modification in patients with ACS are sparse. The subject of this investigation is the implementation and quality of risk factor adjustment in the context of a randomized controlled trial.

**Methods:**

A total of 513 patients in the prematurely terminated, randomized, controlled, multicenter SPACE-2 trial (ISRCTN 78592017) were analyzed within one year after randomization into 3 groups (CEA, CAS, and BMT only) for implementation of prespecified BMT recommendations and lifestyle modifications. Measurement time points were the screening visit and visits after one month (D30), 6 months (M6), and one year (A1). Differences between groups and follow-up visits (FUVs) relative to the screening visit were investigated.

**Findings:**

For all FUVs, a significant increase in statin medication (91% at A1; *p* < 0.0001) was demonstrated to be associated with a significant decrease (*p* < 0.01) in cholesterol levels (median 167 mg/dl at A1) and LDL cholesterol levels (median 93 mg/dl at A1). The lowest cholesterol levels were achieved by patients in the BMT group. Seventy-eight percent of all patients reached predefined target cholesterol levels (< 200 mg/dl), with significantly better rates in the BMT group (*p* = 0.036 at D30). Furthermore, a significant decrease in arterial blood pressure at all FUVs (*p* < 0.05) was associated with a significant increase in antihypertensive medication (96% at A1, *p* < 0.0001). However, only 28% of patients achieved the predefined treatment goal of a systolic blood pressure of ≤ 130 mmHg. Forty-two of a total of 100 smokers at the screening visit quit smoking within one year, resulting in a significant increase in nonsmokers at all FUVs (*p* < 0.0001). Recommended HbA1c levels (< 7%) were achieved in 82% without significant changes after one year. Only 7% of obese (BMI > 25) patients achieved sufficient weight reduction after one year without significant changes at all FUVs (median BMI 27 at A1; *p* = 0.1201). The BMT group showed significantly (*p* = 0.024) higher rates of adequate physical activity than the intervention groups. Furthermore, after one year, the BMT group showed a comparatively significantly better implementation of risk factor modification (77%; *p* = 0.027) according to the treating physician.

**Interpretation:**

SPACE-2 demonstrated sustained improvement in the noninterventional management of vascular risk factors in patients treated in a clinical trial by general practitioners, internists and neurologists. The best implemented treatment targets were a reduction in cholesterol and HbA1c levels. In this context, a significant increase in statin use was demonstrated. Blood pressure control missed its target but was significantly reduced by intensification of antihypertensive medication. Patients on BMT only had better adjusted lipid parameters and were more physically active. However, all groups failed to achieve sufficient weight reduction. Due to insufficient patient recruitment, the results must be interpreted cautiously.

*Trial registration*: ISRCTN Registry, ISRCTN78592017, Registered 16 June 2007, https://www.isrctn.com/search?q=78592017.

**Supplementary Information:**

The online version contains supplementary material available at 10.1186/s42466-021-00153-w.

## Introduction

Patients with asymptomatic carotid artery stenosis (ACS) should be treated with best medical treatment (BMT) or in addition invasively with carotid endarterectomy (CEA) or carotid artery stenting (CAS). Regardless of the chosen treatment option, implementation of BMT and lifestyle modification are crucial for reducing the risk of cerebrovascular events. A cohort study of 2,885,257 individuals between 2003 and 2008 revealed the risk factors for the development of ≥ 50% carotid artery stenosis. Among others, uncontrolled hypertension (odds ratio (OR) 2.0; 95% confidence interval (CI) 2.0–2.0), high cholesterol (OR 1.4; 95% CI 1.4–1.4), diabetes (OR 1.4; 95% CI 1.4–1.4), and smoking (depending on packyears, minimum OR 1.5; 95% CI 1.5–1.6) were significant risk factors [1, 2]. These factors also play an important role in risk prediction models for the detection of ACS [3] and are not only crucial for its development but are also considered general stroke risk factors [4]. Consequently, blood pressure and diabetes control, as well as lipid-lowering medications, are considered important components of BMT [5–7]. Antiplatelet therapy has a lower level of evidence in primary prevention and may be indicated in patients with low bleeding risk and cardiovascular comorbidity [8–10]. Statins, in particular, have shown a significant impact on plaque stabilization and reduction in microembolisms with a consecutive reduced risk of vascular events [11]. Despite the clear evidence for the preventive benefits of statins, they are still underutilized [12, 13]. A dose-dependent increase in mortality with nonadherence has been demonstrated [14]. In addition, smoking cessation, maintenance of a healthy body weight, moderate exercise, and a Mediterranean diet are essential complementary behaviors in the prevention of arterial disease not only of the brain supplying vessels but also in other arterial beds [5–7, 15, 16]. The importance of optimized BMT and continuously monitored and improved lifestyle modifications is considered largely underestimated by both physicians and patients [6]. To illustrate the decrease in annual ipsilateral stroke risk over the years in patients with ACS treated with BMT alone, a 2014 a metanalysis of 41 studies showed a rate of 1.0% in patients recruited between 2000 and 2009 compared to 2.3% when recruited before 2000 (*p* < 0.001). Regarding statin use, the rate was 2.3% in studies with the proportion of patients receiving statins was < 25% versus a rate of 1.2% when ≥ 25% of patients received statins (*p* = 0.009) [17]. Therefore, regardless of the use of interventional or conservative therapy, adequate BMT should be implemented.

We investigated whether sufficient implementation of BMT and lifestyle modification could be achieved within one year in a randomized controlled trial. Factors with sufficient implementation could provide examples of effective management of cerebrovascular risk factors beyond clinical trials.

## Methods

The international randomized, controlled, open, multicenter SPACE-2 study (ISRCTN78592017) recruited 513 patients with ACS of the common and/or internal carotid artery of ≥ 70%^ECST^ [18] ≈ ≥ 50%^NASCET^ [19]. Patients were randomized to three treatment arms: CEA plus BMT (n = 203), CAS plus BMT (n = 197), and BMT alone (n = 113). Enrollment occurred in 36 study centers in Germany, Switzerland, and Austria. Further details and endpoints have been previously published [20]. The SPACE-2 study started in 2009. Due to insufficient recruitment rates, a change in study design was implemented in 2013 [21]. However, a persistently too-low recruitment rate despite protocol modification led to the premature termination of the study in 2014 [22]. One-year interim results of the study were published recently [23]. In this investigation, the data from the first year after randomization were analyzed regarding the implementation of BMT and lifestyle modification. At the screening visit (D0), after 30 days (D30), after 6 months (M6), and after one year (A1), the following examinations were recorded: blood samples with fasting glucose/HbA1c/cholesterol/LDL-cholesterol/HDL-cholesterol and triglyceride levels; systolic and diastolic blood pressure, height, and weight with calculated body mass index (BMI; body mass/square of height); waist-to-hip ratio (WHR; waist circumference/hip circumference). Physical activity over 30 min per week was recorded in three categories: never, 1–2 times, and 3 times or more. Smoking behavior and alcohol consumption were recorded. Medications including antiplatelet, anticoagulant, antihypertensive, lipid-lowering and antidiabetic agents and comorbidities, especially those with vascular effects such as arterial hypertension, diabetes mellitus, coronary artery disease, and hypercholesterolemia, were documented. The modified Rankin scale and National Institute of Health Stroke Scale score were assessed. Extra- and intracranial sonography of the supplying vessels of the brain was performed. Before the start of the study, BMT recommendations were established for all centers and all patient groups according to current treatment guidelines [24] (see Additional file 1: Table S1). Adjustment to more strictly defined target value ranges during the course of the study was at the discretion of the individual centers. After the screening visit, all patients received recommendations for individualized medication, optimization of lifestyle modification (nicotine withdrawal, physical activity, alcohol consumption, healthy diet), and further medical examinations (electrocardiography/echocardiography for cardiac status and ankle-brachial index for diagnosis of peripheral arterial occlusive disease). At each of the follow-up visits, the aforementioned risk factors were reviewed, and the implementation of the recommended treatment strategies was re-examined. An assessment of drug compliance was made for the lipid-lowering preparations by measuring cholesterol levels, for antihypertensives by monitoring blood pressure, and for antidiabetics by monitoring blood glucose parameters. For control of medication, the current medication list of the primary care physician and the information provided by the patient were used as a basis. Drug intolerances or side effects were evaluated, and medication adjustments were initiated if necessary. All submitted documentation of blood pressure values, laboratory parameters or hospitalizations was reviewed. All findings, adaptation of medication and goals for further reduction of vascular risk factors were explained to the patients and documented for the general practitioner. The respective study centers were responsible for providing information material, offering telephone contact to clarify problems, and recommending outpatient measures such as smoking cessation courses or other measures to support compliance.

### Statistical analysis

Analysis of the rates of patients achieving the predefined BMT recommendations, blood sample parameters, blood pressure, BMI, WHR, physical activity, implementation of risk factors as assessed by the treating physician, and detailed medication was performed with the chi-square test or Fisher`s exact test (if cell count < 5) and further with the Mann–Whitney U or Kruskal–Wallis test, respectively. Comparisons of the values at the 30-day, one-month, and one-year visits with the baseline values at the screening visit were performed with McNemar’s test and the Wilcoxon signed-rank test. A *p* value < 0.05 was considered statistically significant. Due to death/missing values, the total number of patients varied between the different variables; therefore, percentages were chosen as the primary measure. Since none of the analyses addressed a main target of the main study, all tests were exploratory. Analyses were performed with STATA/IC 13.0 (College Station, Texas, US).

## Results

### Achievement of BMT targets

See Additional file 1: Table S1 for the definition of targets for the BMT. The percentage achievement of the specified BMT targets at visits D30 to A1 is presented in Table 1. Approximately 70% of all patients did not reach the required threshold values when adjusting their blood pressure. Within one year, only a small improvement in blood pressure control was achieved, with no significant difference between the CEA, CAS and BMT groups. Cholesterol levels < 200 mg/dl were reached in 83% of the BMT group (after 30 days) and improved to 88% (after one year), with a significant difference from the CEA group (between 70%-76%) and the CAS group (between 77 and 79%); (pD30 = 0.036; pM6 = 0.041). Sufficiently lowered LDL cholesterol levels < 130 mg/dl (patients without coronary heart disease (CHD)) and < 100 mg/dl (patients with CHD) were achieved by 70% (CEA at D30) to 83% (BMT M6) of the group members without significant differences between the study groups. Sufficient control of HDL cholesterol could be achieved in 77–81% of patients without significant differences between the groups. Triglyceride target values < 150 mg/dl were achieved in 58–75% of patients, with significantly better results in the BMT group after 6 months but not after one year. In all patients, HbA1c values < 7% were achieved in 81–89% of patients without significant differences between treatment groups. In the CEA group, significantly more patients had quit smoking 30 days after the screening visit (CEA: 10%, CAS: 4%, BMT: 1%; *p* = 0.013). The overall rate of patients who had quit smoking went from 5% at the 30-day visit to another 4% at the 6-month visit and another 2% at the 1-year visit. When considering all patients, a significant increase in nonsmoking was achieved over time (screening visit: 80%, after one year: 85%; *p* < 0.0001; see Additional file 1: Table S2). In the BMT group, patients had significantly more physical activity after one month (BMT: 67%, CEA: 52%, CAS: 49%; *p* = 0.015), with increasing rates in the CAS group but slightly decreasing rates in the BMT group within one year. At the screening visit, 377 (73.0%) of all 513 patients had a BMI ≥ 25. The number of patients initially overweight at the screening visit with subsequent sufficient weight reduction increased nonsignificantly at a low level, from 4% after 30 days to 7% at the one-year visit. For further details, see Table 1.

**Table 1:** Proportion of patients with sufficient implementation of BMT goals; visit D30 to A1; n = 513

	CEA (% [n])	CAS (% [n])	BMT (% [n])	total (%[n])	*p*
**Blood pressure ≤ 130/85 mmHg**^**1**^					
D30	23.5% (44)	26.6% (47)	22.9% (24)	24.5% (115)	*0.772*^*⌐*^
M6	25.4% (45)	30.6% (53)	31.1% (33)	28.7% (131)	*0.461*^*⌐*^
A1	25.7% (44)	29.8% (50)	30.1% (31)	28.3% (125)	*0.638*^*⌐*^
**Cholesterol < 200 mg/dl**					
D30	69.6% (103)	79.2% (114)	83.1% (74)	76.4% (291)	*0.036*^⌐^*
M6	75.8% (116)	76.5% (114)	88.3% (83)	79.0% (313)	*0.041*^⌐^*
A1	74.5% (117)	77.1% (111)	87.5% (77)	78.4% (305)	*0.054*^⌐^
**LDL-cholesterol < 130 mg/dl**^**2**^					
D30	69.9% (100)	75.9% (107)	77.3% (68)	73.9% (275)	*0.372*^⌐^
M6	72.8% (110)	71.9% (105)	82.6% (76)	74.8% (291)	*0.140*^⌐^
A1	75.0% (117)	76.8% (109)	75.9% (66)	75.8% (292)	*0.939*^⌐^
**HDL-cholesterol ≥ 40 mg/dl**					
D30	76.7% (112)	76.8% (109)	80.5% (70)	77.6% (291)	*0.766*^⌐^
M6	76.7% (115)	80.1% (117)	78.5% (73)	78.4% (305)	*0.768*^⌐^
A1	79.7% (122)	77.1% (111)	78.4% (69)	78.4% (302)	*0.857*^⌐^
**Triglycerides < 150 mg/dl**					
D30	64.8% (94)	66.4% (93)	63.6% (56)	65.1% (243)	*0.907*^⌐^
M6	57.9% (88)	66.0% (97)	75.3% (70)	65.1% (255)	*0.021*^⌐^*
A1	58.2% (89)	65.3% (94)	62.5% (55)	61.8% (238)	*0.567*^⌐^
**HbA1c < 7%**					
D30	89.1% (123)	84.9% (118)	77.3% (68)	84.7% (309)	*0.054*^⌐^
M6	84.3% (118)	87.0% (120)	81.3% (74)	84.6% (312)	*0.510*^⌐^
A1	82.4% (126)	82.6% (119)	81.8% (72)	82.3% (317)	*0.408*^⌐^
**Quit smoking if smoker on D0**					
D30	9.5% (13)	4.2% (6)	1.0% (1)	5.3% (20)	*0.013*^±^*
M6	3.9% (5)	4.4% (6)	5.0% (5)	4.4% (16)	*0.927*^⌐^
A1	3.4% (4)	1.6% (2)	0	1.8% (6)	*0.208* ^±^
**Physical activity ≥ 30 min 3–5 times/week**					
D30	52.4% (97)	49.4% (85)	66.7% (70)	54.5% (252)	*0.015*^⌐^*
M6	53.9% (96)	54.7% (94)	62.9% (66)	56.3% (256)	*0.297*^⌐^
A1	47.1% (81)	55.4% (93)	60.4% (61)	53.3% (235)	*0.082*^⌐^
**Sufficient weight reduction**^**3**^** if BMI ≥ 25 on D0**					
D30	5.1% (7)	3.0% (4)	5.7% (4)	4.4% (15)	*0.583*^±^
M6	6.9% (9)	3.8% (5)	6.8% (5)	5.6% (19)	*0.483*^⌐^
A1	7.1% (9)	6.3% (8)	6.8% (5)	6.7% (22)	*0.964*^⌐^

*BMI* body mass index

*Significant

^±^Fisher`s exact test

^⌐^Chi^2^-Test

^1^If comorbidity diabetes mellitus: ≤ 130/80 mmHg

^2^If comorbidity coronary heart disease: < 100 mg/dl

^3^If BMI 25–27.5: Weight reduction to BMI < 25, if BMI > 27.5: 10% weight reduction; D0: Screening visit; D30: Visit after 30 days; M6: Visit after six months; A1: Visit after one year

### Control of risk factors

Relative to the screening visit, a significant reduction in blood pressure (BP, systolic and diastolic) was achieved over time in all patients (median systolic BP D0: 146 mm Hg, D30: 142 mm Hg (*p* = 0.0005), M6: 141 mm Hg (*p* < 0.0001), A1: 140 mm Hg (*p* < 0.0001)). No persistently significant changes in blood glucose or HbA1c levels were observed over time between groups. In terms of lipid parameters, the BMT group had significantly lower cholesterol levels at 1 year (CEA: 174 mg/dl, CAS 167 mg/dl, BMT 164 mg/dl; *p* = 0.0385) and significantly lower LDL cholesterol levels at the 6-month visit (CEA: 98 mg/dl, CAS: 95 mg/dl, BMT: 87 mg/dl; *p* = 0.0286) than the intervention groups. Significant reductions in cholesterol and LDL cholesterol were achieved in all patients at each follow-up visit relative to the screening visit (see Table 2 for details). BMI did not differ significantly between groups. Compared with that at the screening visit, the discrete but significant decrease in BMI at the 30-day visit was not maintained in all patients at the 6-month and 1-year visits, and BMI remained at its median of 27. Patients in the BMT group were significantly more likely to be physically active at 30 days (physical activity 3 times or more per week: CEA: 52%, CAS 49%, BMT 67%; *p* = 0.024). The advantage of physical activity in the BMT group persisted at the 6-month and 1-year visits but with no significant difference and decreasing rates. According to the treating physician's assessment, implementation of risk factor modification was significantly better in the BMT group at 6 months (BMT: 76%, CEA: 68%, CAS: 54%; *p* < 0.001) and at 1 year (BMT: 77%, CEA: 65%, CAS: 61%; *p* = 0.027). For further details, see Table 2.

**Table 2:** Adjustment of vascular risk factors over time and comparison of study groups; n = 513

	CEA	CAS	BMT	total	*p*^*1*^	*p*^*2*^
Systolic BP D0 (median [IQR])^□^	147 (131; 160)	145 (130; 162)	150 (133; 160)	146 (130; 160)	*0.7317*^◊^	
Diastolic BP D0 (median [IQR])^□^	80 (72; 88)	80 (74; 90)	80 (75; 89)	80 (73; 89)	*0.5734*^◊^	
Systolic BP D30 (median [IQR])	142 (131; 160)	140 (130; 154)	141 (130; 160)	142 (130; 160)	*0.2178*^◊^	*0.0005*^⌂^*
Diastolic BP D30 (median [IQR])	80 (71; 85)	80 (71; 87)	80 (70; 89)	80 (71; 86)	*0.9233*^◊^	*0.0160*^⌂^*
Systolic BP M6 (median [IQR])	144.5 (130; 158)	140 (130; 159)	140 (128; 160)	141 (130; 159)	*0.6510*^◊^	< *0.0001*^⌂^*
Diastolic BP M6 (median [IQR])	80 (70; 87)	80 (74; 86)	80 (72; 86)	80 (72; 86)	*0.4862*^◊^	*0.0035*^⌂^*
Systolic BP A1 (median [IQR])	142.5 (130; 158)	140 (130; 156)	140 (130; 150)	140 (130; 157)	*0.6320*^◊^	< *0.0001*^⌂^*
Diastolic BP A1 (median [IQR])	80 (70.5; 85)	80 (72; 85)	80 (70; 85)	80 (71; 85)	*0.8059*^◊^	*0.0001*^⌂^*
Blood glucose (median [IQR]) D0^□^	103.5 (92; 121)	104 (93; 125)	104 (92; 119)	104 (92.5; 122)	*0.8918*^◊^	
Blood glucose (median [IQR]) D30	110.5 (95; 131)	105 (95; 125)	101 (92; 121)	107 (94; 125)	*0.2776*^◊^	*0.1054*^*⌂*^
Blood glucose (median [IQR]) M6	108 (96; 125)	103 (94; 126)	101 (92.5; 123)	104 (94; 125)	*0.3761*^◊^	*0.8950*^*⌂*^
Blood glucose (median [IQR]) A1	105 (93; 124)	105 (93; 124)	105.5 (96.5; 127)	105 (94; 124.5)	*0.6192*^◊^	*0.2191*^*⌂*^
HbA1c (in %; median [IQR]) D0^□^	6 (5.7; 6.45)	6 (5.7; 6.6)	5.9 (5.55; 6.75)	6 (5.7; 6.5)	*0.5940*^◊^	
HbA1c (in %; median [IQR]) D30	5.9 (5.6; 6.4)	5.9 (5.6; 6.4)	5.9 (5.5; 6.6)	5.9 (5.6; 6.4)	*0.7903*^◊^	*0.0025*^⌂^*
HbA1c (in %; median [IQR]) M6	6.1 (5.7; 6.6)	6.0 (5.7; 6.4)	5.9 (5.5; 6.7)	6.0 (5.7; 6.5)	*0.4636*^◊^	*0.7925*^*⌂*^
HbA1c (in %; median [IQR]) A1	6.0 (5.5; 6.5)	5.9 (5.7; 6.5)	6.0 (5.5; 6.7)	6.0 (5.6; 6.5)	*0.9039*^◊^	*0.5419*^*⌂*^
Cholesterol (median [IQR]) D0^□^	181 (153; 209)	173.5 (152; 200)	169.5 (146.5; 192.5)	174 (151; 203)	*0.2619*^◊^	
Cholesterol (median [IQR]) D30	175 (149; 207)	168 (150.5; 193.5)	159 (142; 191)	168.5 (148; 196)	*0.0744*^◊^	*0.0025*^⌂^*
Cholesterol (median [IQR]) M6	176 (149; 199)	170 (150; 194)	161 (143; 175)	168 (147; 194)	*0.0056*^◊^*	*0.0091*^⌂^*
Cholesterol (median [IQR]) A1	174 (150; 202)	167 (143; 198)	164 (140; 184)	167 (146; 194)	*0.0385*^◊^*	*0.0017*^⌂^*
LDL-Cholesterol (median [IQR]) D0^□^	105 (81; 130)	98.5 (80; 126)	97 (80; 117)	99 (80; 123.5)	*0.3559*^◊^	
LDL-Cholesterol (median [IQR]) D30	98 (78; 125)	91 (76; 114)	88 (72; 106)	91 (76; 116)	*0.0985*^◊^	*0.0001*^⌂^*
LDL-Cholesterol (median [IQR]) M6	98 (75; 119)	95 (80; 115)	87 (72; 103)	92 (75; 113)	*0.0286*^◊^*	*0.0006*^⌂^*
LDL-Cholesterol (median [IQR]) A1	98 (78; 118)	93 (72; 116)	87 (69; 108)	93 (73; 116)	*0.1164*^◊^	*0.0001*^⌂^*
HDL-Cholesterol (median [IQR]) D0^□^	48 (40; 62)	49 (40; 56.5)	49 (41.5; 57.5)	49 (41; 58)	*0.8799*^◊^	
HDL-Cholesterol (median [IQR]) D30	50 (40; 60)	48 (40; 60)	50 (41; 59)	49 (41; 60)	*0.6703*^◊^	*0.8535*^*⌂*^
HDL-Cholesterol (median [IQR]) M6	49 (40; 60)	49 (41; 60)	48 (41; 56)	49 (41; 59)	*0.8393*^◊^	*0.6249*^*⌂*^
HDL-Cholesterol (median [IQR]) A1	48 (41; 59.5)	49 (40; 58.5)	49.5 (41.5; 60)	49 (41; 59.5)	*0.9204*^◊^	*0.0592*^*⌂*^
Triglycerides (median [IQR]) D0^□^	131 (98; 182.5)	128.5 (92; 184)	120 (91; 181)	126.5 (94; 182)	*0.6626*^◊^	
Triglycerides (median [IQR]) D30	121 (92, 207)	114 (87; 174)	121 (87; 181)	119 (88; 181)	*0.5671*^◊^	*0.0893*^*⌂*^
Triglycerides (median [IQR]) M6	133.5 (94; 192)	124 (90; 164)	107 (78; 143)	122.5 (89; 173.5)	*0.0123*^◊^*	*0.0581*^*⌂*^
Triglycerides (median [IQR]) A1	136 (97; 192)	112.5 (88; 182)	132 (91; 173)	127 (91; 183)	*0.0920*^◊^	*0.5763*^*⌂*^
BMI D0 (median [IQR])^□^	27 (25; 30)	27 (25; 30)	27 (24; 29)	27 (25; 30)	*0.3567*^◊^	
BMI D30 (median [IQR])	27 (25; 30)	27 (25; 30)	26.5 (24; 29)	27 (25; 30)	*0.1906*^◊^	*0.0001*^⌂^*
BMI M6 (median [IQR])	27 (25; 30)	27.5 (25; 30)	27 (24; 29)	27 (25; 30)	*0.2135*^◊^	*0.3891*^*⌂*^
BMI A1 (median [IQR])	27 (25; 29.5)	27 (25; 30)	26 (24; 30)	27 (25; 30)	*0.5458*^◊^	*0.1201*^*⌂*^
WHR D0 (median [IQR])	0.97 (0.93; 1.02)	0.98 (0.94; 1.04)	0.99 (0.95; 1.03)	0.98 (0.94; 1.03)	*0.1514*^◊^	
WHR D30 (median [IQR])	0.98 (0.93; 1.03)	0.98 (0.94; 1.03)	0.99 (0.93; 1.03)	0.98 (0.93; 1.03)	*0.7593*^◊^	*0.8432*^*⌂*^
WHR M6 (median [IQR])	0.98 (0.95; 1.02)	0.97 (0.93; 1.02)	1.00 (0.94; 1.03)	0.98 (0.94; 1.02)	*0.2148*^◊^	*0.3083*^*⌂*^
WHR A1 (median [IQR])	0.99 (0.95; 1.02)	0.98 (0.92; 1.03)	0.99 (0.94; 1.03)	0.98 (0.94; 1.02)	*0.6700*^◊^	*0.7363*^*⌂*^
Physical activity > 30 min/week D30 (% [n])						
None	24.9% (46)	26.2% (45)	11.4% (12)	22.3% (103)	*0.024* ^⌐^*	
1–2 times	22.7% (42)	24.4% (42)	21.9% (23)	23.2% (107)		
3 times or more	52.4% (97)	49.4% (85)	66.7% (70)	54.5% (252)		
Physical activity > 30 min/week M6 (% [n])						
None	20.2% (36)	25.6% (44)	17.1% (18)	21.5% (98)	*0.267*^⌐^	
1–2 times	25.8% (46)	19.8% (34)	20.0% (21)	22.2% (101)		
3 times or more	53.9% (96)	54.7% (94)	62.9% (66)	56.3% (256)		
Physical activity > 30 min/week A1 (% [n])						
None	19.8% (34)	23.2% (39)	16.8% (17)	20.4% (90)	*0.069*^⌐^	
1–2 times	33.1% (57)	21.4% (36)	22.8% (23)	26.3% (116)		
3 times or more	47.1% (81)	55.4% (93)	60.4% (61)	53.3% (235)		
Implementation of RFM at D30 (% [n])	74.4% (131)	63.9% (108)	68.0% (70)	69.0% (309)	*0.104*^⌐^	
Implementation of RFM at M6 (% [n])	67.8% (116)	53.7% (88)	76.0% (79)	64.5% (283)	< 0.001*	
Implementation of RFM at A1 (% [n])	64.7% (110)	60.8% (101)	76.8% (76)	66.0% (287)	*0.027*^⌐^*	

BP: Blood pressure; BMI: Body mass index; WHR: Waist-hip ratio; all parameters of lipid metabolism and glucose in mg/dl; LDL: Low-density lipoprotein; HDL: High-density lipoprotein; RFM: Risk factor modification

^*^Significant

^⌐^Chi^2^-Test

^◊^Kruskal–Wallis test

^*⌂*^Wilcoxon signed-rank test

^□^Already published [23]; D30: Visit 30 days after screening visit; M6: Visit 6 months after screening visit; A1: Visit one year after screening visit

^1^testing CEA vs. CAS vs. BMT

^2^testing total value D30/M6/A1 vs. screening visit

### Medication

Compared with the screening visit, significantly more patients were treated with statin medication after 30 days (D0: 77%, D30: 88%; *p* < 0.0001). This significant difference was maintained at subsequent visits (M6: 90%, A1: 91%; *p* < 0.0001). After the screening visit, patients in the BMT group had higher but statistically nonsignificant rates of statin medication use (Table 3).

**Table 3:** Proportion of patients on statin medication in each study group over time; n = 513

Visit	CEA	CAS	BMT	total	*p*^*1*^	*p*^*2*^
Screening^□^	80.3% (163)	73.6% (145)	78.8% (89)	77.4% (397)	*0.257*^*⌐*^	
D30	87.6% (163)	86.7% (156)	91.5% (97)	88.1% (416)	*0.456*^*⌐*^	< *0.0001*^^^*
M6	91.7% (166)	86.9% (152)	93.5% (101)	90.3% (419)	*0.131*^*⌐*^	< *0.0001*^^^*
A1	92.5% (160)	87.6% (149)	93.2% (96)	90.8% (405)	*0.190*^*⌐*^	< *0.0001*^^^*

^*^Significant

^⌐^Ch^i2^-test

^^^McNemar test

^□^Already published [23]; D30: Visit 30 days after screening visit; M6: Visit 6 months after screening visit; A1: Visit o ne year after screening visit

^1^testing CEA vs. CAS vs. BMT

^2^testing rate of statin medication on D30/M6/A1 vs. screening visit

There were no significant differences in antiplatelet medication at any visit relative to the screening visit, except for higher rates of clopidogrel and lower rates of ASA plus dipyridamole in the CAS group because of dual antiplatelet therapy with ASA and clopidogrel before stenting and 1 month afterward. Rates of antiplatelet therapy ranged from 97 to 99%. Patients who did not receive antiplatelet therapy had anticoagulation or short-term ASA interruption due to trauma. Patient anticoagulant use was between 2 and 5%. Most likely, due to changes in the study protocol (after 2013, anticoagulation was no longer an exclusion criterion), there was significantly more use of anticoagulants at low levels during follow-up (D0: 2.3%, A1: 4.7%; *p* = 0.0072). Compared with that at the screening visit, the rate of antihypertensives increased significantly at each visit (D0: 87%, D30: 93% (*p* = 0.0015), M6: 94% (*p* = 0.0002), A1: 96% (*p* < 0.0001)). Beta-blockers were mainly used (54–58%), followed by ACE inhibitors (49–54%), diuretics (38–47%), calcium antagonists (26–33%) and AT-II antagonists (26–30%). ACE inhibitors were used significantly more often at 1 month and at 1 year relative to the screening visit. There was also a significant increase in the use of diuretics, calcium antagonists, and beta-blockers. Antidiabetic agents were administered to 25–27% of patients, without significant rates of increase during follow-up. For details on the prescriptions of the above drug groups, see Additional file 1: Table S3.

### Effect of statin medication on lipid levels—intake control

Intake of the prescribed statin medication seems plausible, as cholesterol and LDL cholesterol levels differed significantly (*p* < 0.0001) between patients on and off statin medication at 30 days, 6 months, and at 1 year. LDL cholesterol levels were reduced to 90 mg/dl or less in statin users. HDL cholesterol levels were higher in the statin user group, but the difference was not significant (Additional file 1: Table S4a–c). No significant association between statin use or cholesterol levels and vascular outcome events was demonstrated.

## Discussion

Within 1 year, 513 patients with asymptomatic carotid stenosis in the SPACE-2 study were analyzed for the implementation of the BMT recommendations. Documentation time points were visits 30 days, 6 months, and 1 year after the screening visit. Although the recommended blood pressure of at least ≤ 130/85 mm Hg was achieved in only 25–30% of patients, a significant increase in antihypertensive medication rates from 87 to 96% with a consecutive significant reduction in blood pressure from a median of 146/80 mmHg to 140/80 mmHg was demonstrated. One conclusion could be that, even if the recommended treatment goals are not achieved, following the target of given BMT recommendations leads to a significant reduction in blood pressure. Treatment of hypertension is a high priority because it is highly effective in preventing ischemic stroke and is the largest modifiable risk factor for stroke, accounting for one-third of strokes in industrialized countries [5]. Another extremely relevant parameter of stroke prophylaxis is a sufficient adjustment of lipid parameters. Statins have a plaque-stabilizing effect [11] and thus play an important role in stroke prevention [5]. A meta-analysis showed that statin treatment increased over time: 67% of trials that ended enrollment from 2000 onward reported that at least 25% of their participants received statins, compared with 4% of trials that ended enrollment earlier (*p* < 0.001), with a significantly lower incidence of ipsilateral stroke in trials in which ≥ 25% of patients used statins [17]. Therefore, consistently adjusted statin medication is one of the main components of BMT. In the Asymptomatic Carotid Surgery Trial (ACST), the proportion of patients on lipid-lowering therapy was reported to be less than 10% in 1993 and increased to more than 80% by the end of follow-up in 2006–08, with lower rates of stroke and death in patients on lipid-lowering therapy [25]. The implementation of recommendations for pharmacological therapy in the routine treatment of ACS was demonstrated in a retrospective single center analysis from 2002 to 2014 with a significant increase in the use of antiplatelet therapy (72% to 96%) and statins (13% to 79%) [26]. Due to increased statin use, a risk reduction for cerebral infarction has also been shown in symptomatic carotid stenosis, further emphasizing the importance of medical therapy [27]. However, even in this controlled trial setting, only approximately 75% of patients achieved sufficient adjustment of the required cholesterol levels. Meanwhile, since the start of the SPACE-2 trial in 2009, the cutoff values for LDL cholesterol were further reduced (< 100 mg/dl without CHD, < 70 mg/dl with CHD) [10] or even more strictly reduced to a target of 55 mg/dl and a reduction in LDL of > 50% of the initial value for patients in secondary cardiovascular prevention and for patients at very high risk [16]. Because of these stricter targets, recent guidelines recommend the use of more potent CSE inhibitors, the additional administration of ezetimibe, or even PCSK9 inhibitors [28]. Regarding statin medication implementation, we found a significantly increased rate from 77 to 91% in all patients during the course of the study, with consecutive significant reductions in cholesterol levels (median 174 mg/dl to 167 mg/dl) and LDL cholesterol levels (median 99 mg/dl to 93 mg/dl). Adequate medication compliance could be plausibly demonstrated with a significant reduction in cholesterol and LDL cholesterol levels in patients on statin medication relative to those without. These results are consistent with data from a meta-analysis of 86 randomized controlled trials, which also demonstrated significantly reduced LDL cholesterol, increased rates of statin prescription and improved statin adherence [13]. This study also showed improved LDL cholesterol reduction over time in studies published after 2012. However, this meta-analysis did not identify a single implementation strategy or group of implementation strategies with superior impact to others [13]. In the SPACE-2 trial, 42% of all patients smoking at baseline had quit smoking after one year. In the context of this study, this is above the known real-world rate of approximately 20% within a comparable period [29]. However, SPACE-2 also showed a decrease in motivation over the course of the study, with the rate of patients who quit smoking falling from 5% after one month to 2% after one year.

### Was the BMT group more motivated?

The highest rates of statin medication use were found in the BMT group (but with no significant difference from the intervention groups), resulting in significantly lower (LDL)-cholesterol levels in some cases. In addition, patients in the BMT group, in particular, met the prespecified targets for cholesterol levels (with a significant difference from the intervention groups) and LDL/HDL cholesterol levels (without a significant difference). The BMT patients had the best values for lifestyle modification in the form of sufficient physical activity in accordance with the predefined treatment goals. After 6 months and after 1 year, the BMT group showed significantly better implementation of risk factor management as assessed by the treating physician. The intensified optimization of vascular risk factors might possibly have been motivated by the "fear" of progression of stenosis or ischemic events, whereas interventionally treated patients felt "safer" after a reduction in stenosis. On the other hand, fewer patients in the BMT group quit smoking, and the rates of adequate weight loss decreased over time. Regarding this inadequate weight reduction and given a recommendation rate for diet of only 54% and a recommendation rate for sufficient physical activity of 53% after 1 year, instructions for physical activity and weight reduction should have been emphasized to a greater degree in our study.

The main limitation of the SPACE-2 trial was the premature termination of randomization due to insufficient patient recruitment and a resulting sample size that was too small. Moreover, the statistical concept of this manuscript allows only exploratory, not a confirmatory, analyses. The statistical analyses must therefore be interpreted with caution. Another limitation is the measurement of blood pressure. Since this was performed only once at each visit, a valid interpretation of continuous blood pressure parameters is not possible. The unsatisfactory results in weight reduction may be explained by inadequately implemented nutritional counseling. Taking into account the documentation effort within the scope of the study, measures that were implemented or recommended, such as smoking cessation courses, dietary counseling or fitness training programs, were not recorded. Therefore, a further analysis of the reasons for not achieving the prevention goals was not feasible.

With regard to the analyzed collective, it can be assumed that the patients participating in this study had higher level to reduce vascular risk factors than the collective found in everyday practice. Nevertheless, the improvement in risk modification demonstrated in SPACE-2 through clear goal setting (avoidance of stenosis progression/occurrence of stenosis-related symptoms) and regular visit contacts can also be implemented in a nonstudy setting. Regarding the sustainable effect of the different therapy methods, it could be argued that the interventional methods (CEA/CAS) show a more stable effect after their one-time application than the continuously controlled medical therapy. We showed in this controlled study that BMT can also be maintained at a high level or even improved. In this context, consistent medical therapy must be supplemented with lifestyle modification and continuous motivation for weight reduction and physical activity.

## Conclusion

The SPACE-2 trial demonstrated sustained improvement in noninterventional vascular risk management in patients with ACS treated by general practitioners, internists and neurologists. Target values for cholesterol and HbA1c were best achieved. In this context, a significant increase in statin intake was relevant. Blood pressure control missed the prespecified target, but blood pressure was significantly reduced by intensification of antihypertensive medication. Patients receiving BMT alone without interventional therapy had significantly better adjusted lipid parameters and, in some cases, more physical activity. However, no treatment group achieved sufficient weight reduction. Our data emphasize the importance of individualized treatment and standardized follow-up protocols in all patients at high risk for vascular or cerebral complications. Patients should be informed of detailed treatment goals and of the consequences if they are missed. A comprehensive patient- and family-centered approach might be more efficient [30]. Due to insufficient patient recruitment, the results must be interpreted with caution.

## Supplementary Information

**Additional file 1**.** Table S1**: Prespecified treatment goals for vascular risk factors in SPACE-2.** Table S2**: Nonsmoking rates and avoidance of obesity over time.** Table S3**: Medication from screening visit to one year.** Table S4** a/b/c: Lipid level as a function of statin intake after 30 days, 6 months and one year.
