# Supplementary material for: Successful implementation of best medical treatment for patients with asymptomatic carotid artery stenosis within a randomized controlled trial (SPACE-2)
Source: Neurol Res Pract. 2021 Oct 19;3:62. doi: 10.1186/s42466-021-00153-w (PMC8524978; doi:10.1186/s42466-021-00153-w)
Supplement: Supplementary file 1 — Additional file 1. Table S1: Prespecified treatment goals for vascular risk factors in SPACE-2. Table S2: Nonsmoking rates and avoidance of obesity over time. Table S3: Medication from screening visit to one year. Table S4 a/b/c: Lipid level as a function of statin intake after 30 days, 6 months and one year. [file 42466_2021_153_MOESM1_ESM.pdf]

Successful implementation of best medical treatment for patients with asymptomatic carotid artery stenosis within a randomized controlled trial (SPACE-2).

Supplemental Tables

Table S1: Prespecified treatment goals for vascular risk factors in SPACE-2 (already published[1]).

**Risk factor**	**Treatment target**
Smoking	cessation
Fasting total cholesterol	<200mg/dl
LDL cholesterol (no CHD)	<130mg/dl
LDL cholesterol (with CHD)	<100mg/dl
HDL cholesterol	≥40mg/dl
Triglycerides	<150mg/dl
Overweight BMI 25-27.5	weight loss until BMI <25
Overweight BMI >27.5	10% weight loss
Blood pressure (without diabetes)	≤130/85 mm Hg
Blood pressure (with diabetes)	≤130/80 mm Hg
Diabetes mellitus	HbA1c<7%
Physical activity	At least 30 min. of physical activity 3x/week
Alcohol consumption	male <30g/day, female <15g/day

CHD: coronary heart disease; BMI: body mass index; LDL: low density lipoprotein; HDL: high density lipoprotein.

Table S2: Nonsmoking rates and avoidance of obesity over time; n=513.

	CEA (% [n])	CAS (%[n])	BMT (%[n])	total (%[n])	p^2	p^3
Packyears[1] (median [IQR])	24.3 (11.25; 40)	31.5 (15.6; 49)	30 (11.5; 44)	28.5 (13; 45)	*0.108*◊	
Non-smokers						
D0	77.2% (152)	84.1% (164)	78.6% (88)	80.2% (404)	*0.202*⌐	
D30	82.0% (109)	85.8% (121)	76.9% (70)	82.2% (300)	*0.224*⌐	<0.0001^*
M6	80.7% (109)	88.3% (128)	83.0% (83)	84.7% (322)	*0.209*⌐	<0.0001^*
A1	83.3% (120)	89.6% (129)	81.1% (77)	85.1% (326)	*0.145*⌐	<0.0001^*
BMI <25						
D0	24.0% (47)	23.6% (46)	28.8% (32)	24.9% (125)	*0.553*⌐	
D30	24.5% (45)	23.7% (42)	33.3% (34)	26.1% (121)	*0.171*⌐	0.8318^
M6	26.1% (46)	22.4% (39)	29.5% (31)	25.5% (116)	*0.406*⌐	1.0000^
A1	27.1% (46)	25.7% (43)	25.5% (26)	26.2% (115)	*0.947*⌐	0.2962^

*significant; ◊Kruskal–Wallis test; ⌐Chi^2-Test; ^McNemar's test; BMI: Body mass index; [1]screening visit; D0: Screening visit; D30: Visit after 30 days; M6: Visit after six months; A1: Visit after one year; [2]testing CEA vs. CAS vs. BMT; [3]testing total value D30/M6/A1 vs. D0 visit.

Table S3: Medication from screening visit to one year, comparison between groups and over time; n=513.

	CEA (% [n])	CAS (%[n])	BMT (%[n])	total (%[n])	*p*[3]	*p*[4]
**Antiplatelets**						
D0□	95.6% (194)	96.4% (190)	98.2% (111)	96.5% (495)	0.749$^{\pm}$	
D30	98.9% (184)	100% (180)	98.1% (104)	99.2% (468)	0.172$^{\pm}$	0.0225$^{\wedge}$*
M6	98.3% (178)	98.9% (173)	97.2% (105)	98.3% (456)	0.594$^{\pm}$	0.7905$^{\wedge}$
A1	96.0% (166)	98.2% (167)	96.1% (99)	96.9% (432)	0.447$^{\pm}$	1.0000$^{\wedge}$
**ASA[1]**						
D0□	80.3% (163)	83.8% (165)	86.7% (98)	83.0% (426)	0.325^{-}	
D30	86.6% (161)	97.2% (175)	83.0% (88)	89.8% (424)	<0.001$^{\pm}$*	0.0002$^{\wedge}$*
M6	82.9% (150)	88.6% (155)	81.5% (88)	84.7% (393)	0.187^{-}	1.0000$^{\wedge}$
A1	79.2% (137)	85.9% (146)	78.6% (81)	81.6% (364)	0.188^{-}	0.3961$^{\wedge}$
**ASA[1] + Dipyridamol**						
D0□	4.4% (9)	2.0% (4)	3.5% (4)	3.3% (17)	0.393$^{\pm}$	
D30	2.2% (4)	0	3.8% (4)	1.7% (8)	0.022$^{\pm}$*	0.0703$^{\wedge}$
M6	2.8% (5)	1.1% (2)	3.7% (4)	2.4% (11)	0.358$^{\pm}$	1.0000$^{\wedge}$
A1	2.3% (4)	0	3.9% (4)	1.8% (8)	0.027$^{\pm}$*	0.2891$^{\wedge}$
**Clopidogrel**						
D0□	16.3% (33)	20.3% (40)	16.8% (19)	17.9% (92)	0.539^{-}	
D30	15.6% (29)	86.1% (155)	18.9% (20)	43.2% (204)	<0.001^{-}*	<0.0001$^{\wedge}$*
M6	16.0% (29)	30.3% (53)	14.8% (16)	21.1% (98)	0.001^{-}*	0.1945$^{\wedge}$
A1	17.9% (31)	21.2% (36)	17.5% (18)	19.1% (85)	0.668^{-}	0.9088$^{\wedge}$
**Others**						
D0□	1.0% (2)	1.5% (3)	0.9% (1)	1.2% (6)	0.878$^{\pm}$	
D30	1.1% (2)	3.3.% (6)	2.8% (3)	2.3% (11)	0.355$^{\pm}$	0.1250$^{\wedge}$
M6	1.7% (3)	1.7% (3)	0.9% (1)	1.5% (7)	1.000$^{\pm}$	0.6875$^{\wedge}$
A1	2.9% (5)	1.8% (3)	1.0% (1)	2.0% (9)	0.692$^{\pm}$	0.4531$^{\wedge}$
**Anticoagulants**						
D0□	2.0% (4)	2.5% (5)	2.7% (3)	2.3% (12)	0.870$^{\pm}$	
D30	1.6% (3)	4.0% (7)	1.9% (2)	2.6% (12)	0.389$^{\pm}$	1.0000$^{\wedge}$
M6	1.7% (3)	2.3% (4)	3.7% (4)	2.4% (11)	0.504$^{\pm}$	0.8036$^{\wedge}$
A1	4.0% (7)	5.3% (9)	4.9% (5)	4.7% (21)	0.859^{-}	0.0072$^{\wedge}$*
**Heparin**						
D0□	0.5% (1)	1.5% (3)	1.8% (2)	1.2% (6)	0.518$^{\pm}$	
D30	0.5% (1)	1.7% (3)	0	0.8% (4)	0.364$^{\pm}$	0.7539$^{\wedge}$
M6	0.6% (1)	0	0	0.2% (1)	1.000$^{\pm}$	0.1250$^{\wedge}$
A1	0	1.2% (2)	0	0.4% (2)	0.198$^{\pm}$	0.6875$^{\wedge}$
**Vitamin-K-antagonists**						
D0□	0.5% (1)	0.5% (1)	0.9% (1)	0.6% (3)	1.000$^{\pm}$	
D30	0	1.1% (2)	1.9% (2)	0.8% (4)	0.125$^{\pm}$	1.0000$^{\wedge}$
M6	0.6% (1)	0.6% (1)	0.9% (1)	0.6% (3)	1.000$^{\pm}$	1.0000$^{\wedge}$
A1	1.7% (3.)	3.5% (6)	1.9% (2)	2.5% (11)	0.588$^{\pm}$	0.0215$^{\wedge}$*
**Others**						
D0□	1.5% (3)	0.5% (1)	0	0.8% (4)	0.546$^{\pm}$	
D30	1.1% (2)	1.1% (2)	0	0.8% (4)	0.684$^{\pm}$	1.0000$^{\wedge}$
M6	0.6% (1)	1.7% (3)	2.8% (3)	1.5% (7)	0.270$^{\pm}$	0.1250$^{\wedge}$
A1	1.7% (3)	1.8% (3)	2.9% (3)	2.0% (9)	0.760$^{\pm}$	0.0391$^{\wedge}$*
**Antihypertensives**						
D0□	85.7% (174)	87.3% (172)	90.3% (102)	87.3% (448)	0.667^{-}	
D30	94.0% (173)	90.4% (160)	93.4% (99)	92.5% (432)	0.393^{-}	0.0015$^{\wedge}$*
M6	94.5% (171)	92.6% (162)	93.5% (100)	93.5% (433)	0.766^{-}	0.0002$^{\wedge}$*
A1	95.4% (165)	95.3% (161)	96.1% (99)	95.5% (425)	1.000$^{\pm}$	<0.0001$^{\wedge}$*
**ACE[2]-inhibitos**						
D0□	49.8% (101)	49.7% (98)	46.9% (53)	49.1% (252)	0.867^{-}	
D30	56.6% (105)	55.0% (99)	49.1% (52)	54.2% (256)	0.459^{-}	0.0014$^{\wedge}$*
M6	58.0% (105)	53.1% (93)	43.5% (47)	52.8% (245)	0.057^{-}	0.0869$^{\wedge}$
A1	55.5% (96)	56.5% (96)	47.6% (49)	54.0% (241)	0.319^{-}	0.0363$^{\wedge}$*
**Angiotensin-II-receptor-antagonists**						
D0□	27.1% (55)	22.3% (44)	31.0% (35)	26.1% (134)	0.230^{-}	
D30	30.6% (57)	22.8% (41)	36.8% (39)	29.0% (137)	0.034^{-}*	0.2430$^{\wedge}$
M6	29.8% (54)	24.0% (42)	38.0% (41)	29.5% (137)	0.043^{-}*	0.1114$^{\wedge}$
A1	27.7% (48)	25.9% (44)	38.8% (40)	29.6% (132)	0.060^{-}	0.0869$^{\wedge}$
**Diuretics**						
D0□	45.3% (92)	30.5% (60)	38.1% (43)	38.0% (195)	0.009^{-}*	

D30	50.5% (94)	36.1% (65)	42.5% (45)	43.2% (204)	0.020¯*	0.0125^*
M6	54.7% (99)	38.9% (68)	37.0% (40)	44.6% (207)	0.002¯*	0.0080^*
A1	55.5% (96)	40.0% (68)	42.7% (44)	46.6% (208)	0.011¯*	0.0004^*
**Calcium antagonists**						
D0▫	29.6% (60)	23.9% (47)	24.8% (28)	26.3% (135)	0.396¯	
D30	34.9% (65)	28.3% (51)	27.4% (29)	30.7% (145)	0.272¯	0.0137^*
M6	37.6% (68)	28.6% (50)	31.5% (34)	32.8% (152)	0.185¯	0.0009^*
A1	38.7% (67)	27.1% (46)	30.1% (31)	32.3% (144)	0.060¯	0.0154^*
**Beta blockers**						
D0▫	51.7% (105)	54.8% (108)	55.8% (63)	53.8% (276)	0.738¯	
D30	55.9% (104)	56.7% (102)	54.7% (58)	55.9% (264)	0.950¯	0.3604^
M6	58.6% (106)	57.1% (100)	54.6% (59)	57.1% (265)	0.804¯	0.2370^
A1	60.7% (105)	56.5% (96)	56.3% (58)	58.1% (259)	0.671¯	0.0414^*
**Others**						
D0▫	9.4% (19)	10.2% (20)	8.0% (9)	9.4% (48)	0.817¯	
D30	11.3% (21)	10.0% (18)	8.5% (9)	10.2% (48)	0.745¯	0.7359^
M6	9.9% (18)	8.0% (14)	10.2% (11)	9.3% (43)	0.771¯	1.0000^
A1	11.0% (19)	11.2% (19)	8.7% (9)	10.5% (47)	0.793¯	0.5682^
**Lipid-lowering-drugs**						
D0▫	82.3% (167)	78.7% (155)	85.0% (96)	81.5% (418)	0.455¯	
D30	89.7% (165)	88.8% (159)	94.3% (99)	90.4% (423)	0.294¯	<0.0001^*
M6	92.2% (166)	88.0% (154)	94.4% (102)	91.1% (422)	0.145¯	<0.0001^*
A1	92.5% (160)	88.2% (150)	94.2% (97)	91.3% (407)	0.185¯	<0.0001^*
**Statin**						
D0▫	80.3% (163)	73.6% (145)	78.8% (89)	77.4% (397)	0.257¯	
D30	87.6% (163)	86.7% (156)	91.5% (97)	88.1% (416)	0.456¯	<0.0001^*
M6	91.7% (166)	86.9% (152)	93.5% (101)	90.3% (419)	0.131¯	<0.0001^*
A1	92.5% (160)	87.6% (149)	93.2% (96)	90.8% (405)	0.190¯	<0.0001^*
**Other**						
D0▫	6.9% (14)	6.6% (13)	7.1% (8)	6.8% (35)	0.986¯	
D30	2.7% (5)	6.7% (12)	7.5% (8)	5.3% (25)	0.118¯	0.2101^
M6	4.4% (8)	6.3% (11)	5.6% (6)	5.4% (25)	0.735¯	0.3877^
A1	5.2% (9)	6.5% (11)	5.8% (6)	5.8% (26)	0.882¯	0.6636^
**Antidiabetic drugs**						
D0▫	24.1% (49)	25.4% (50)	31.0% (35)	26.1% (134)	0.422¯	
D30	21.9% (40)	25.8% (46)	28.3% (30)	24.8% (116)	0.439¯	0.2500^
M6	23.5% (42)	26.4% (46)	29.6% (32)	26.0% (120)	0.508¯	0.3750^
A1	23.1% (40)	27.8% (47)	30.1% (31)	26.5% (118)	0.397¯	0.3438^
**Insulin**						
D0▫	9.4% (19)	7.6% (15)	9.7% (11)	8.8% (45)	0.760¯	
D30	9.7% (18)	8.3% (15)	9.4% (10)	9.1% (43)	0.897¯	1.0000^
M6	10.5% (19)	9.1% (16)	11.1% (12)	10.1% (47)	0.849¯	0.0654^
A1	9.2% (16)	8.8% (15)	9.7% (10)	9.2% (41)	0.970¯	0.1460^
**Oral antidiabetics**						
D0▫	18.7% (38)	21.3% (42)	23.9% (27)	20.9% (107)	0.544¯	
D30	16.1% (30)	22.2% (40)	21.7% (23)	19.7% (93)	0.288¯	0.5488^
M6	17.1% (31.)	22.3% (39)	23.1% (25)	20.5% (95)	0.355¯	0.1185^
A1	17.9% (31)	24.1% (41)	24.3% (25)	21.7% (97)	0.296¯	0.5413^

*significant; ±Fisher`s exact test; ¯Chi^2-Test; ^McNemar's test; BMI: Body mass index; [1]Acetylsalicyclic acid; [2]Angiotensin converting enzyme; [3]testing CEA vs. CAS vs. BMT; [4]testing rate of medication (total) in D30/M6/A1 vs. screening visit; D0: Screening visit; ▫ already published[1]; D30: Visit after 30 days; M6: Visit after six months; A1: Visit after one year.

Table S4a: Lipid level as a function of statin intake after 30 days; n=513.

	statin use	CEA	CAS	BMT	total	*p*[◊]
Cholesterol (median [IQR])	yes	169 (143; 193)	164 (148; 184)	156 (136; 180)	164 (144; 187)	*<0.0001*[‡]*
	no	226 (210; 248)	199.5 (178; 231)	216 (201.5; 228)	216 (197; 237)	
LDL-Cholesterol (median [IQR])	yes	91 (75; 112)	89 (74; 110)	84 (70.5; 102)	88 (73; 107)	*<0.0001*[‡]*
	no	149 (125; 161)	117 (97; 153)	144.5 (137; 161)	138 (106; 159)	
HDL-Cholesterol (median [IQR])	yes	51 (45; 61)	43 (35.5; 46.5)	48.5 (42.5; 54)	49 (40; 60)	*0.6006*[‡]
	no	49 (40; 60)	48 (41; 60)	50 (41; 59)	47 (42; 57)	
Triglycerides (median [IQR])	yes	118.5 (88; 190)	111 (86; 167)	112 (82.5; 185.5)	115 (86; 177)	*0.0251*[‡]*
	no	143 (97; 177)	141 (104; 220)	154 (111.5; 174)	143.5 (100; 207)	

*significant; [‡]Mann–Whitney U test for all patients; [◊]for total values; all values in mg/dl.

Table S4b: Lipid level as a function of statin intake after 6 months; n=513.

	statin use	CEA	CAS	BMT	total	*p*[◊]
Cholesterol (median [IQR])	yes	172 (145; 195)	169 (152; 191)	160 (142; 172)	167 (145; 188)	*<0.0001*[‡]*
	no	231.5 (198; 245)	198.5 (182; 244)	209 (157; 243)	211.5 (189; 243.5)	
LDL-Cholesterol (median [IQR])	yes	94 (73; 113)	93 (80; 110.5)	85 (72; 98)	90 (75; 110)	*<0.0001*[‡]*
	no	138 (119; 151)	121 (112; 157)	140 (104; 178)	137 (115; 154)	
HDL-Cholesterol (median [IQR])	yes	49 (40; 60)	49 (43; 61)	49 (41; 58)	49 (41; 60)	*0.0508*[‡]
	no	47 (40; 58)	41.5 (36; 54)	45.5 (41; 56)	44 (39; 56)	
Triglycerides (median [IQR])	yes	129 (92; 190)	122.5 (90; 161)	107 (78; 144)	121 (88; 173)	*0.0748*[‡]
	no	146 (76; 198)	159.5 (95; 274)	141.5 (113; 162)	153 (95; 259)	

*significant; [‡]Mann–Whitney U test for all patients; [◊]for total values; all values in mg/dl.

Table S4c: Lipid level as a function of statin intake after 1 year; n=513.

	statin use	CEA	CAS	BMT	total	*p*[◊]
Cholesterol (median [IQR])	yes	167 (149; 196)	165 (140.5; 187)	162 (140; 181)	164.5 (145; 186.5)	*<0.0001*[‡]*
	no	220 (202; 232)	206 (180; 227)	241 (201; 253)	214 (182.5; 231.5)	
LDL-Cholesterol (median [IQR])	yes	91 (75; 115)	89.5 (70; 116)	85.5 (69; 103)	88 (72; 111)	*<0.0001*[‡]*
	no	137 (121; 154)	130 (103; 156)	147 (142; 163)	139.5 (111.5; 155)	
HDL-Cholesterol (median [IQR])	yes	48 (41; 59)	49 (40; 58)	50 (43; 60)	49 (41; 59)	*0.2557*[‡]
	no	47 (40; 59)	42 (36; 51)	50.5 (44; 73)	44 (37; 57)	
Triglycerides (median [IQR])	yes	135 (97; 186)	111 (85; 171)	132 (89.5; 170.5)	126 (90; 174)	*0.1994*[‡]
	no	124 (89; 197)	154 (94; 218)	166.5 (106; 218)	131 (94; 215)	

*significant; [‡]Mann–Whitney U test for total; [◊]for total values; all values in mg/dl.

References

1. Reiff T, Eckstein HH, Mansmann U, Jansen O, Fraedrich G, Mudra H, et al. Angioplasty in asymptomatic carotid artery stenosis vs. endarterectomy compared to best medical treatment: One-year interim results of SPACE-2. Int J Stroke. 2020;15 6:638-49; doi: 10.1177/1747493019833017.
